# Chlorophyll fluorescence imaging accurately quantifies freezing damage and cold acclimation responses in Arabidopsis leaves

**DOI:** 10.1186/1746-4811-4-12

**Published:** 2008-05-27

**Authors:** Britta Ehlert, Dirk K Hincha

**Affiliations:** 1 Max-Planck-Institut für Molekulare Pflanzenphysiologie, Am Mühlenberg 1, D-14476 Potsdam, Germany

## Abstract

**Background:**

Freezing tolerance is an important factor in the geographical distribution of plants and strongly influences crop yield. Many plants increase their freezing tolerance during exposure to low, nonfreezing temperatures in a process termed cold acclimation. There is considerable natural variation in the cold acclimation capacity of Arabidopsis that has been used to study the molecular basis of this trait. Accurate methods for the quantitation of freezing damage in leaves that include spatial information about the distribution of damage and the possibility to screen large populations of plants are necessary, but currently not available. In addition, currently used standard methods such as electrolyte leakage assays are very laborious and therefore not easily applicable for large-scale screening purposes.

**Results:**

We have performed freezing experiments with the Arabidopsis accessions C24 and Tenela, which differ strongly in their freezing tolerance, both before and after cold acclimation. Freezing tolerance of detached leaves was investigated using the well established electrolyte leakage assay as a reference. Chlorophyll fluorescence imaging was used as an alternative method that provides spatial resolution of freezing damage over the leaf area. With both methods, LT_50 _values (i.e. temperature where 50% damage occurred) could be derived as quantitative measures of leaf freezing tolerance. Both methods revealed the expected differences between acclimated and nonacclimated plants and between the two accessions and LT_50 _values were tightly correlated. However, electrolyte leakage assays consistently yielded higher LT_50 _values than chlorophyll fluorescence imaging. This was to a large part due to the incubation of leaves for electrolyte leakage measurements in distilled water, which apparently led to secondary damage, while this pre-incubation was not necessary for the chlorophyll fluorescence measurements.

**Conclusion:**

Chlorophyll fluorescence imaging is an alternative method to accurately determine the freezing tolerance of leaves. It is quick and inexpensive and the system could potentially be used for large scale screening, allowing new approaches to elucidate the molecular basis of plant freezing tolerance.

## Background

Freezing tolerance is an important factor in determining the natural geographic distribution of plant species and the growth area and yield of many crop plants. Many plants from temperate and cold climates, including several important crop species, are able to increase their freezing tolerance in response to low, nonfreezing temperatures in a process termed cold acclimation (see [1,2] for comprehensive reviews). Plant freezing tolerance is a multigenic, quantitative trait and cold acclimation in the well-studied model plant *Arabidopsis thaliana *involves expression changes in hundreds of genes [3-6] and changes in the cellular content of a large fraction of measurable metabolites [4,5,7-9].

Attempts to understand the genetic and molecular basis of complex quantitative traits in plants have in recent years focussed on the analysis of natural genetic variation. *A. thaliana *is a geographically widely spread species and it has been shown that different accessions have sufficient genetic variability to allow investigations of genotype × environment interactions (see [10,11] for reviews). Such natural variation has also been shown for Arabidopsis freezing tolerance and cold acclimation capacity [4,12].

An important prerequisite for the analysis of such phenotypic variability is the ability to accurately quantify plant freezing tolerance and cold acclimation responses. It is generally agreed in the field that the primary targets of freezing damage in plants are cellular membranes (see [13,14] for comprehensive reviews). Consequently, besides whole plant survival, the determination of electrolyte leakage from plant tissues after freezing and thawing, using conductivity measurements, has been the most frequently used method reported in the literature (see e.g. [15] and references therein). This method mainly monitors the ability of the plasma membrane to function as a semi-permeable barrier towards intracellular ions, but the intactness of the vacuole, as the major storage compartment for inorganic ions, may also impact the measurements.

In addition, chloroplast membranes may also be damaged during freezing and thawing, as shown by electron microscopy [16]. Functionally, this results in an inactivation of photosynthesis, which is related to the inactivation of linear electron transport (see [17] for a review). In isolated thylakoid membranes, this could be related to the loss of plastocyanin from the thylakoid lumen [18-20]. The release of the electron transport protein plastocyanin was also observed during freezing of detached spinach leaves and was shifted to lower freezing temperatures by a period of cold acclimation [21-23]. The inactivation of photosynthesis in spinach leaf discs during a freeze-thaw cycle could also be observed by chlorophyll fluorescence measurements [24]. In addition, chlorophyll fluorescence measurements were successfully employed to detect freezing damage in other plant species, mainly in conifer needles (reviewed in [25]).

All three methods have been used to determine freezing damage in leaves, but entail different problems. The plastocyanin measurements require highly specific antibodies and are very laborious and time consuming. The electrolyte leakage measurements are less time consuming than the immunological assays, but screening of larger populations of plants would still require considerable efforts. Chlorophyll fluorescence measurements are quick and inexpensive, however, it is unclear whether freezing damage develops homogeneously over the whole leaf area, making measurements at a single, small point of the leaf surface potentially prone to large errors. Since all three methods give no spatial resolution, an alternative method is needed.

In the present paper we show that chlorophyll fluorescence imaging is such a method, as it provides spatial resolution and can rapidly quantify freeze-thaw damage. The fluorescence images clearly showed spatial heterogeneity of freeze-thaw injury in partially damaged leaves and integration of the fluorescence signals over the whole leaf area allowed quantitative comparisons between different Arabidopsis accessions and between acclimated and nonacclimated plants.

## Results

For the chlorophyll fluorescence imaging measurements, fluorescence induction curves upon illumination of dark adapted leaves were recorded, where fluorescence is emitted from the chlorophyll a of photosystem II (see [26-29] for detailed reviews of the technology). From these induction curves, the maximum quantum use efficiency of photosystem II can be calculated as F_V_/F_M_. In healthy, nonstressed leaves F_V_/F_M _usually has a value of around 0.83 [30]. This value declines under the influence of stress factors. Changes in F_V_/F_M _are thus a sensitive and rapid indicator of the functional state of the photosynthetic apparatus [28,31].

**Figure 1:**
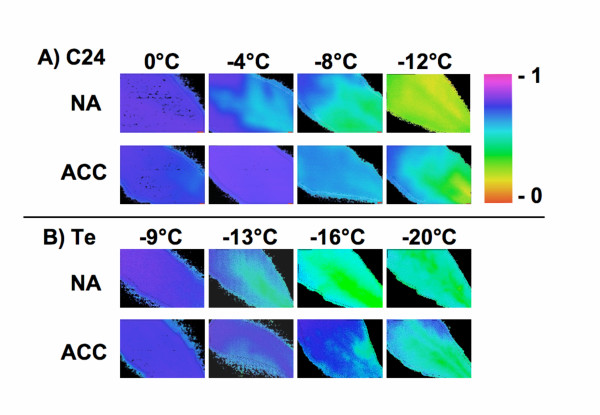
Freezing damage in Arabidopsis leaves visualized by chlorophyll fluorescence imaging. The panels show false color images of the chlorophyll fluorescence yield (F_V_/F_M_) of detached leaves from the accessions C24 **(A) **and Te **(B) **after freezing to the indicated temperatures at 2°C/h and slow thawing on ice. Plants were harvested either directly from a greenhouse under nonacclimating conditions (NA), or after an additional 14 days of cold acclimation at 4°C (ACC).

Figure 1 shows false color images of chlorophyll fluorescence yield (F_V_/F_M_) from detached Arabidopsis leaves either incubated at 0°C as unfrozen controls or frozen to different temperatures and subsequently thawed and dark adapted before measurement. To test whether this method provides reliable information on the physiological state of the leaves, we compared leaves from plants grown under nonacclimating conditions (NA) with plants that were additionally kept at 4°C for 14 days of cold acclimation (ACC). It has been shown before that such an acclimation treatment results in increased freezing tolerance in Arabidopsis, as measured by whole plant survival or electrolyte leakage assays. In addition, we have compared two accessions, C24 and Te, that have been shown by electrolyte leakage measurements to differ both in their NA and ACC freezing tolerance [4].

The images clearly show increased inactivation of photosynthesis with decreasing freezing temperatures in all cases. Leaves from both accessions increase in freezing tolerance during cold acclimation, as clearly shown by the shift of the onset of changes in the coloration (i.e. in F_V_/F_M_) to lower temperatures in the acclimated as compared to the nonacclimated leaves. Finally, the difference between the two accessions that was shown by electrolyte leakage measurements in a previous study [4] was also evident at the chloroplast level (note the different temperatures in Fig. 1A and 1B). The figure shows no images for the 0°C controls in Te, as these were indistinguishable from those taken after freezing to -9°C or the 0°C controls in C24.

In addition, the images provide spatial information about the development of freezing damage in the leaves. Damage started in the basal parts of the leaves and then spread successively throughout the leaf lamina. This was not directly related to ice crystallisation events, as in all samples crystallization was seeded at -1°C. Therefore, all leaves were already completely frozen before the first signs of damage appeared. This is most striking in the Te leaves, where no change in F_V_/F_M _was apparent down to -9°C (Fig. 1B).

**Figure 2:**
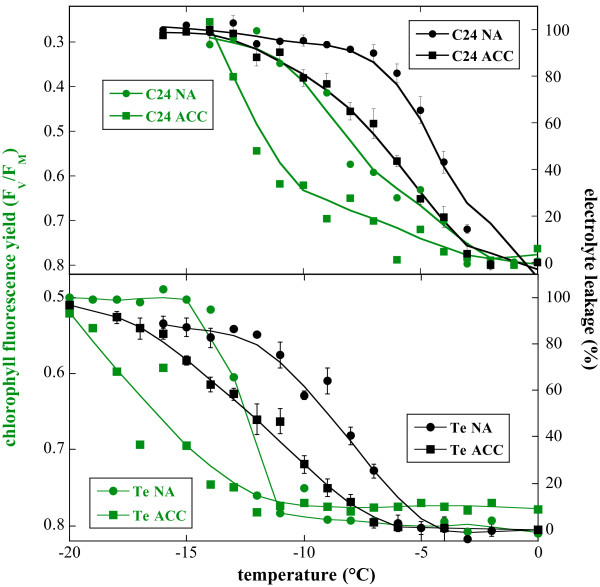
Quantitative comparison of freezing damage to detached Arabidopsis leaves determined either by chlorophyll fluorescence imaging (F_V_/F_M_; compare Fig. 1) or by electrolyte leakage measurements. Leaves for the freezing experiments were harvested from plants of the accessions C24 (upper panel) or Te (lower panel) either before (NA) or after cold acclimation (ACC). Error bars for electrolyte leakage data denote SEM from three to five replica. Chlorophyll fluorescence was measured on single samples. Measurements of chlorophyll fluorescence and electrolyte leakage were performed on parallel plants in the same experiments. The F_V_/F_M _axis has been inverted to allow for a more direct comparison between chlorophyll fluorescence and electrolyte leakage data.

For a comparison with electrolyte leakage data, F_V_/F_M _was integrated over the imaged leaf area to obtain a quantitative measure of freezing damage for each leaf. Figure 2 shows a direct comparison with electrolyte leakage values obtained from parallel samples in the same experiments. The expected differences in the electrolyte leakage curves between leaves from NA and ACC plants and between C24 and Te can be clearly observed. Interestingly, in both accessions F_V_/F_M _in the unfrozen controls was slightly lower in the ACC than in the NA leaves, possibly indicating small photoinhibitory effects during cold acclimation.

**Figure 3:**
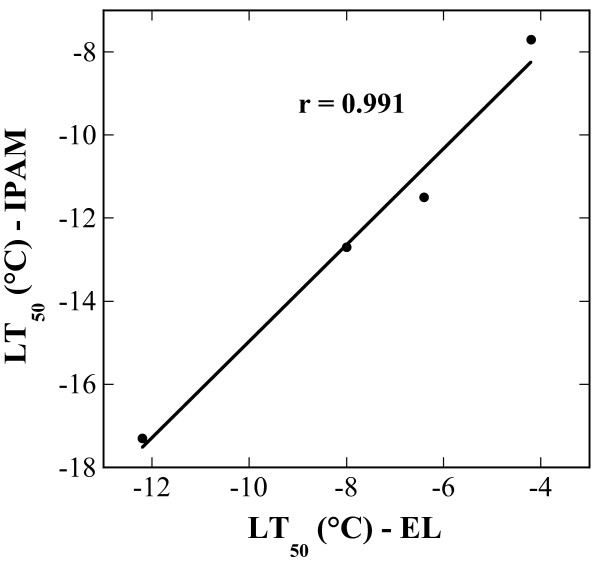
Correlation between LT_50 _values obtained by electrolyte leakage measurements (LT_50 _– EL) or by chlorophyll fluorescence imaging (LT_50 _– IPAM). LT_50 _values were determined from the curves shown in Fig. 2 and can be found in Table 1. The line was fitted by least squares linear regression analysis and the correlation coefficient (r) is shown in the panel.

It can also be seen that the chlorophyll fluorescence imaging and electrolyte leakage data gave a similar picture, but that the curves for F_V_/F_M _are shifted to lower temperatures. In fact, there was a linear correlation between the values obtained by the two different methods for all samples at all temperatures, with a correlation coefficient of 0.78 and a correlation p-value below 0.0001. More importantly, however, the calculated LT_50 _values indicate that the relative shifts between NA and ACC, and between C24 and Te plants are similar (Table 1). Correlation analysis (Fig. 3) reveals a tight linear correlation between the LT_50 _values derived from the two data sets, indicating the utility of chlorophyll fluorescence imaging as an alternative method to quantify freezing tolerance in leaves.

**Table 1:** LT_50 _values calculated from the electrolyte leakage (EL) and F_V_/F_M _(IPAM) curves shown in Fig. 2.

**Plants**	**LT_50 _(°C) – EL**	**LT_50 _(°C) – IPAM**
**C24 NA**	-4.2	-7.7
**C24 ACC**	-6.4	-11.5
**Te NA**	-8.0	-12.7
**Te ACC**	-12.2	-17.3

The observed differences between electrolyte leakage and chlorophyll fluorescence data (Fig. 2) could indicate that the different membrane systems that the two methods report on significantly differ in their freezing tolerance and/or that technical differences between the two methods are responsible for the divergent results. The main difference in sample treatment before measurement was that for the electrolyte leakage measurements the leaves have to be incubated in distilled water to extract the ions lost from the cells into the intercellular spaces during freezing and thawing, while chlorophyll fluorescence measurements can be performed directly after thawing. The incubation in distilled water could lead to secondary damage to the leaves, especially when the cells had been destabilized by a prior freeze-thaw cycle. Since it was  not possible to omit the incubation step in the electrolyte leakage protocol, we instead compared chlorophyll fluorescence data in leaves that were either pretreated with a 24-h incubation in distilled water, or used directly after thawing.

**Figure 4:**
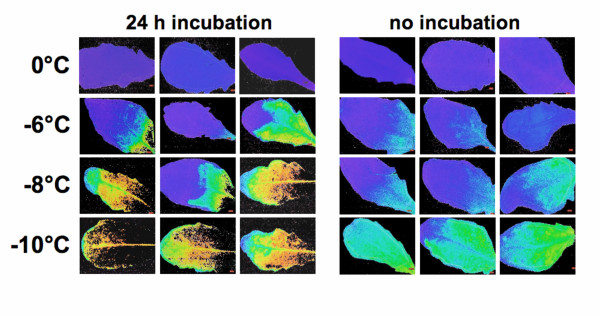
False color images of chlorophyll fluorescence yield (F_V_/F_M_) of Arabidopsis leaves (accession C24 after cold acclimation) after freezing to different temperatures. For the color scale, see Fig. 1. At each temperature, images from three replicate leaves are shown. Leaves were either imaged directly after thawing (no incubation), or after 24 h of incubation in distilled water at 4°C (24 h incubation), corresponding to the treatment of leaves prior to electrolyte leakage measurement.

**Figure 5:**
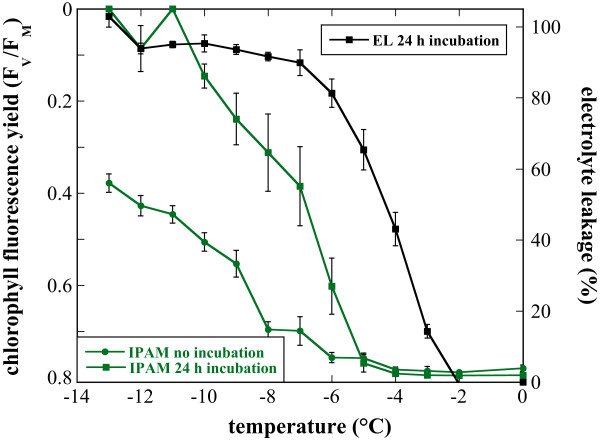
Quantitative comparison of freezing damage to detached Arabidopsis leaves determined by chlorophyll fluorescence imaging (F_V_/F_M_; compare Fig. 1) and electrolyte leakage measurements. The corresponding false color chlorophyll fluorescence images are shown in Fig. 4. Leaves were either imaged directly after thawing (IPAM no incubation), or after 24 h of incubation in distilled water at 4°C (IPAM 24 h incubation and EL 24 h incubation). Error bars denote the SEM from three replicate measurements. The F_V_/F_M _axis has been inverted to allow comparison with the electrolyte leakage data.

This comparison clearly shows that the pre-incubation resulted in additional damage to the leaves after a freeze-thaw cycle (Fig. 4). While the images of the unfrozen controls appeared identical before and after the incubation in distilled water, frozen-thawed leaves were less stable during this incubation and sometimes even completely lost any fluorescence signal. A quantitative analysis of the images (Fig. 5) showed a shift in F_V_/F_M _with pre-incubation in distilled water that brought the curves obtained from electrolyte leakage and chlorophyll fluorescence imaging measurements closer together, but did not completely abolish the differences. Also, linear least squares correlation analysis showed that F_V_/F_M _values and electrolyte leakage values were linearly correlated, but that the correlation was improved when the leaves were incubated in distilled water before the measurements (r = 0.84, p = 0.0003) compared to samples without incubation (r = 0.73, p = 0.0049). This indicates that the experimental treatment rather than biological reasons account for the a large part of the differences between the results obtained with the two methods. However, an intrinsically higher resistance of the photosynthetic apparatus towards freezing damage can also not be completely excluded.

## Discussion

Freezing damage in Arabidopsis and several other plant species has mainly been investigated either by scoring whole plants for survival, or by measuring electrolyte leakage from seedlings or detached leaves. While survival measurements allow no conclusions about specific sites of injury, electrolyte leakage is generally supposed to monitor primarily the intactness of the plasma membrane as a diffusion barrier. Figures 1 and 2 show that chlorophyll fluorescence imaging can be used as an alternative method to assess freezing damage in leaves and that results obtained with both methods are linearly correlated (Fig. 3), allowing quantitative comparisons between datasets. Chlorophyll fluorescence imaging offers the additional advantage of providing spatial information about damage, which no other currently employed method can provide. This showed in our experiments that freezing damage was indeed not homogeneously distributed over the whole leaf area. This emphasizes that point measurements of chlorophyll fluorescence would be highly error prone. Why the basal part of the leaves showed a decrease of F_V_/F_M _at milder freezing temperatures than the upper part, is not clear at present. Since all samples were inoculated with ice at -1°C, differential ice crystallization is an unlikely reason for this pattern, especially at temperatures as low as -20°C in leaves from acclimated Te plants (Fig. 1). Also, careful measurements with thermocouples showed that there were no significant temperature gradients in the part of the test tubes that contained the leaves during freezing (data not shown). Another possible explanation could be that older cells are less freezing tolerant than younger cells. This would be in agreement with the finding that also older leaves as a whole are less freezing tolerant than younger leaves [32].

The spatial resolution provided by chlorophyll fluorescence imaging could in addition be of particular interest in transgenic approaches with promoters that direct expression e.g. to the vascular tissue and where tissue specific protection may otherwise be overlooked, or where the ectopically expressed protein is directed specifically to the chloroplasts [33]. The chlorophyll fluorescence images also indicate that point measurements with a conventional chlorophyll fluorometer are prone to large errors, as fluorescence yield in partially damaged leaves would depend strongly on the location of the point of measurement.

It has often been proposed that the plasma membrane is the primary site of freezing damage in plant cells [14]. Our data provide circumstantial support for this assumption. In fact, even when the fluorescence data were corrected for the effects of pre-incubation of the leaves in distilled water, that is unavoidable in electrolyte leakage assays, both methods yielded comparable but not identical results, indicating that chloroplasts are probably damaged in a slightly lower temperature range than the plasma membrane during freezing.

## Conclusion

Current methods to determine plant freezing tolerance are either only semi-quantitative, such as scoring plants for survival after freezing and thawing, or do not allow high-throughput measurements, such as the electrolyte leakage assays. However, it is possible to adapt the chlorophyll fluorescence imaging system to be used on small seedlings on 96-well plates [31] that allow large scale screening approaches to be used e.g. on mutant collections or recombinant inbred line populations. This approach could in principle also be useful for freezing tolerance measurements, allowing new approaches to elucidate the molecular basis of plant freezing tolerance through large-scale quantitative genetics.

## Methods

### Plant material

We used *Arabidopsis thaliana *plants from the accessions C24 and Tenela (Te) [4]. Plants were grown in soil in a greenhouse at 16 h day length with light supplementation to reach at least 200 μE m^-2 ^s^-1 ^and a temperature of 20°C during the day, 18°C during the night until bolting (compare [15]). For cold acclimation, plants were transferred to a 4°C growth cabinet at 16 h day length with 90 μE m^-2 ^s^-1 ^for an additional 14 days.

### Freezing experiments

Freezing damage was determined as electrolyte leakage after freezing of detached leaves to different temperatures as described in detail in previous publications [4,15]. Briefly, leaves were frozen in pools of three at a rate of 2°C/h to the temperatures indicated in the figures. Parallel samples were kept on ice as unfrozen controls. Frozen samples were thawed slowly on ice. All samples were then immersed in distilled water and placed on a shaker for 16 h at 4°C. Electrolyte leakage was determined as the ratio of conductivity measured in the water before and after boiling the samples. The LT_50 _(temperature of 50% electrolyte leakage) was calculated as the LOGEC50 value of sigmoidal curves fitted to the leakage values using the software GraphPad Prism.

For chlorophyll fluorescence imaging, detached Arabidopsis leaves were frozen and thawed in the same manner as leaves used for the electrolyte leakage assays. In independent experiments chlorophyll fluorescence data in leaves directly after thawing or pretreated with a 24-h incubation in distilled water were compared. Chlorophyll fluorescence imaging was performed as described by Schreiber et al. [34]. Leaves were adapted for 20 min in the dark before measuring the chlorophyll fluorescence with the IPAM Fluorometer (Walz, Germany). With an image processing software (imagewin) false color images of leaf chlorophyll fluorescence yield (F_V_/F_M_) were taken. For quantitative analysis, F_V_/F_M _was integrated over the whole leaf area and the integrated data from leaves frozen to different temperatures were used to calculate LT_50 _values analogous to those derived from the electrolyte leakage measurements.

## List of abbreviations

F_M_: maximal fluorescence from dark-adapted leaves; F_V_: variable fluorescence from dark-adapted leaves; LT_50_: temperature where 50% electrolyte leakage or reduction in F_V_/F_M _occurred

## Authors' contributions

BE participated in the design of the study, performed all experiments and analyzed the data, DKH conceived of the study, participated in its design and data analysis and drafted the manuscript. Both authors read and approved the final manuscript.
